# The Characteristics and Quality of Mobile Phone Apps Targeted at Men Who Have Sex With Men in China: A Window of Opportunity for Health Information Dissemination?

**DOI:** 10.2196/12573

**Published:** 2019-03-27

**Authors:** Guoli Yang, Jian Long, Dan Luo, Shuiyuan Xiao, Atipatsa Chiwanda Kaminga

**Affiliations:** 1 Department of Social Medicine and Health Management, Xiangya School of Public Health, Central South University Changsha China; 2 Department of Emergency, 3rd Xiangya Hospital, Central South University Changsha China; 3 Department of Mathematics and Statistics, Mzuzu University Mzuzu Malawi; 4 Department of Epidemiology and Health Statistics, Xiangya School of Public Health, Central South University Changsha China

**Keywords:** mobile application, homosexuality, male, quality, character

## Abstract

**Background:**

A number of mobile phone apps has been developed specifically for men who have sex with men (MSM). We will call these mobile phone apps *MSM apps* for simplicity. At present, the characteristics and quality and purpose of these MSM apps are unclear.

**Objective:**

The aim of this study was to objectively and comprehensively evaluate the characteristics and quality of the MSM apps to assess whether they disseminated health information among the MSM in China.

**Methods:**

We searched 2 dominant mobile phone app stores (Apple Store and Android Market) to obtain MSM apps using the keywords *MSM, gay, lesbian, gay, bisexual*, and *transgender (LGBT)*, *男同，同志，男男性接触者，同性恋，基友*, and *双性恋*. Apps were excluded if they did not have a Chinese language interface or if their target population was not MSM. Basic information about the eligible apps for this study, such as app name, app store category, and date of last update was gathered from the specified app stores. The quality of apps was rated by 2 independent raters using Mobile App Rating Scale (MARS). The intraclass correlation coefficient (ICC) between raters was computed as a measure for interrater reliability of the MARS. All analyses were conducted using SPSS version 20.0 (SPSS Inc).

**Results:**

A total of 575 apps were reviewed between September 15, 2018 and September 30, 2018, out of which, 532 apps were excluded. Finally, 43 apps were included. Of the 43 apps, 16 were from the Apple Store, 10 were from Android Market, and 17 were available in both app stores. In addition, 39 out of 43 apps were for social and sexual networking, whereas 10 contained sexual health information, for example, HIV/sexually transmitted diseases knowledge, HIV test, and condom use. The average rating was 4 stars. The number of downloads for 21 apps exceeded 10,000. A total of 31 apps had acceptable quality (as defined by a MARS score of >3), with functionality as the highest scoring domain, followed by information quality, esthetics, and engagement. Interrater reliability was excellent for the overall mean app quality scores (ICC=.946; 95% CI 0.904-0.970) and the subjective quality scores (ICC=.910; 95% CI 0.841-0.950).

**Conclusions:**

By reviewing the available apps, we found that MSM apps are popular. The majority of MSM apps are for dating, whereas few of them contain HIV prevention and health information. The overall quality of the apps is acceptable. The utilization of mobile phone technologies is a promising way for delivering HIV prevention messages to MSM. We recommend that researchers and app developers should work together to disseminate health information for MSM via mobile technologies.

## Introduction

### Background

In recent years, smartphone use has been growing rapidly worldwide. For example, in 2015, the global smartphone users were 1.91 billion, and the figure increased to 2.16 billion in 2016 [1]. In China, there were about 0.6 billion smartphone users in 2016, and the number is increasing year by year [2]. Mobile phone apps are computer programs designed to run on smartphones, tablet computers, and other mobile devices. The symbol *app* will be used to mean mobile phone app. Smartphone apps provide a new platform for information distribution and networking. Global mobile app downloads were 197 billion in 2017 versus 149 billion in 2016. By 2021, the total number of app downloads would jump to a stunning 352 billion [3].

In the vast app market, a number of smartphone apps have been developed specifically for men who have sex with men (MSM). These apps will be referred to as *MSM apps* for simplicity. MSM apps have a variety of purposes: social and sexual networking (gay apps) [4], life or entertainment, and research or health education. Traditionally, dating among MSM has been taking place in fixed physical locations such as parks and entertainment facilities (eg, gay-friendly bars or pubs, bathhouses, and saunas) [5,6]. Nonetheless, the gay app is quickly replacing traditional locations as the preferred site to find sexual partners. In 2014, gay apps (eg, Tinder and Grindr) had 90 million users per month worldwide [7]. In a study of 1342 Chinese MSM, 40.6% MSM had used at least one gay app [4]. In a sample of 369 MSM, 60% of MSM reported they used the gay app every day [8]. MSM app users are often young, single, and self-identified as gay [4]. For some MSM, smartphone apps like Grindr, Hornet, FindFred, Growlr, and Scruff have become a part of their daily life [9]. Many MSM used these channels for reasons other than sex seeking, including entertainment and socializing [10,11]. In China, a large proportion of MSM face severe stigma; therefore, they remain closeted. This new mode of communication is subtle and prudent, making it easy for MSM to avoid conflict with heterosexual hegemony in the traditional Chinese culture [12]. In addition, the virtual social platform made by MSM apps strengthens their self-identity and provides emotional support for homosexual identity [13].

MSM apps also provide a vital window of opportunity for health-related organizations to help MSM protect their health, given the rapid spread of HIV/sexually transmitted diseases (STDs) among MSM [14]. Some special apps have been developed by researchers to conduct behavioral intervention among the MSM [15,16]. For example, some MSM apps offer sexual health information, which lets MSM know where the nearest HIV testing center is, where they can obtain postexposure prophylaxis if they have been exposed to HIV, and other health promotion messages [9].

### Objectives

There is a mass of MSM apps available in the Chinese market. However, the characteristics and quality of these apps are unclear. The aim of this study was to objectively and comprehensively evaluate the characteristics (including categories, audience, functional characteristics, contents, and popularity) and quality of MSM apps to assess whether these apps disseminate health information. Understanding this information could help guide the growing body of mobile phone–based health promotion efforts to guard the health of the users of MSM apps in China.

## Methods

### Search and Inclusion and Exclusion Criteria

Android operating systems (82.03%) and iOS (13.20%) are the 2 most dominant mobile phone operating systems, which together account for more than 95% of the Chinese mobile phone market [2]. Therefore, 1 iPhone (Apple Inc) and 1 Android phone (Xiaomi; MI Corporation, Beijing, China) were used to search apps from the Apple Store and Android Market, respectively. Search terms were either in English or in Chinese. English search terms were *MSM, gay, lesbian, gay, bisexual*, and *transgender (LGBT)*, whereas Chinese search terms were *男同，同志，男男性接触者，同性恋，基友*, and *双性恋*. Apps were excluded if they did not have a Chinese language interface or if their target population was not MSM.

### Search Procedures

**Figure 1:**
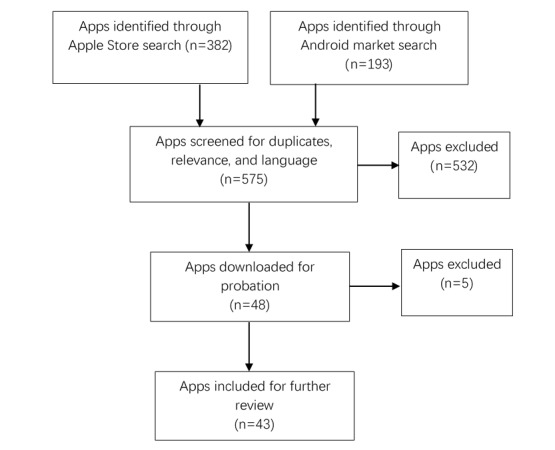
Search and screen flow for men who have sex with men apps.

**Figure 2:**
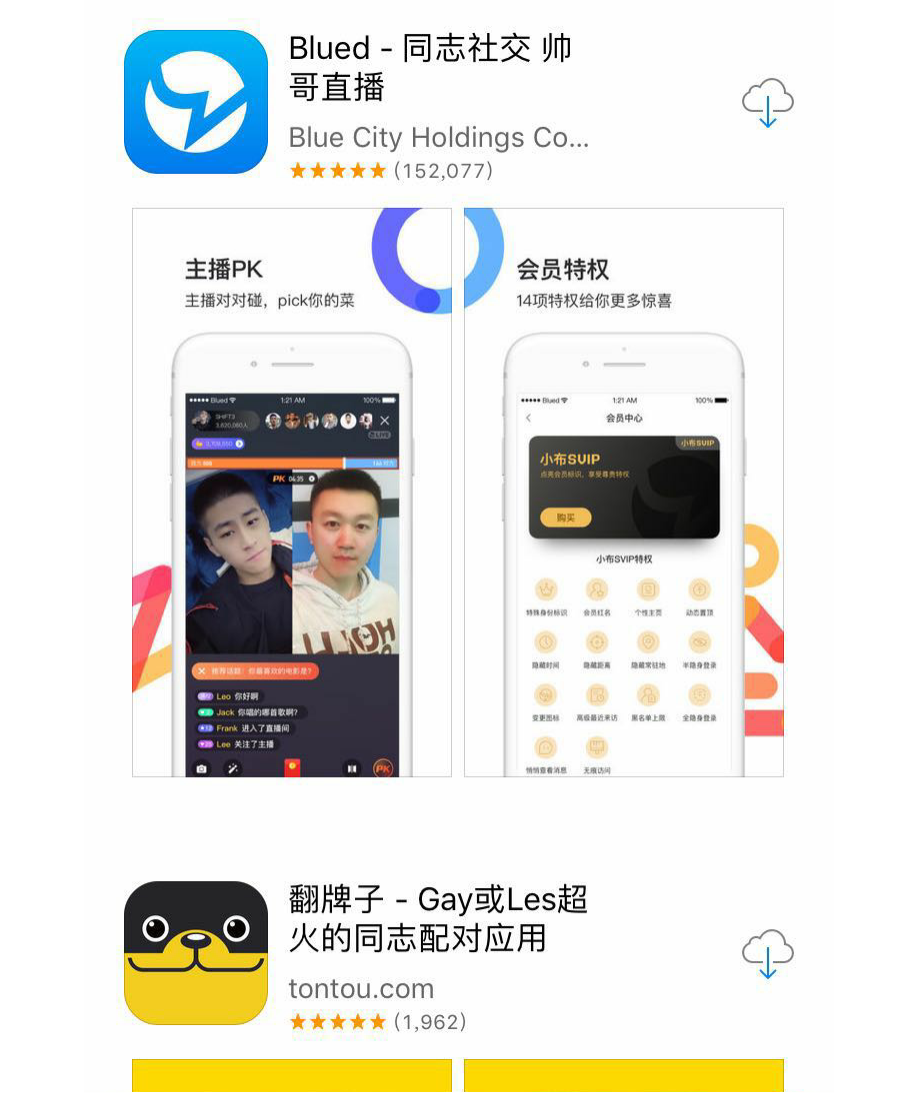
Some examples of MSM Apps for iPhone.

**Figure 3:**
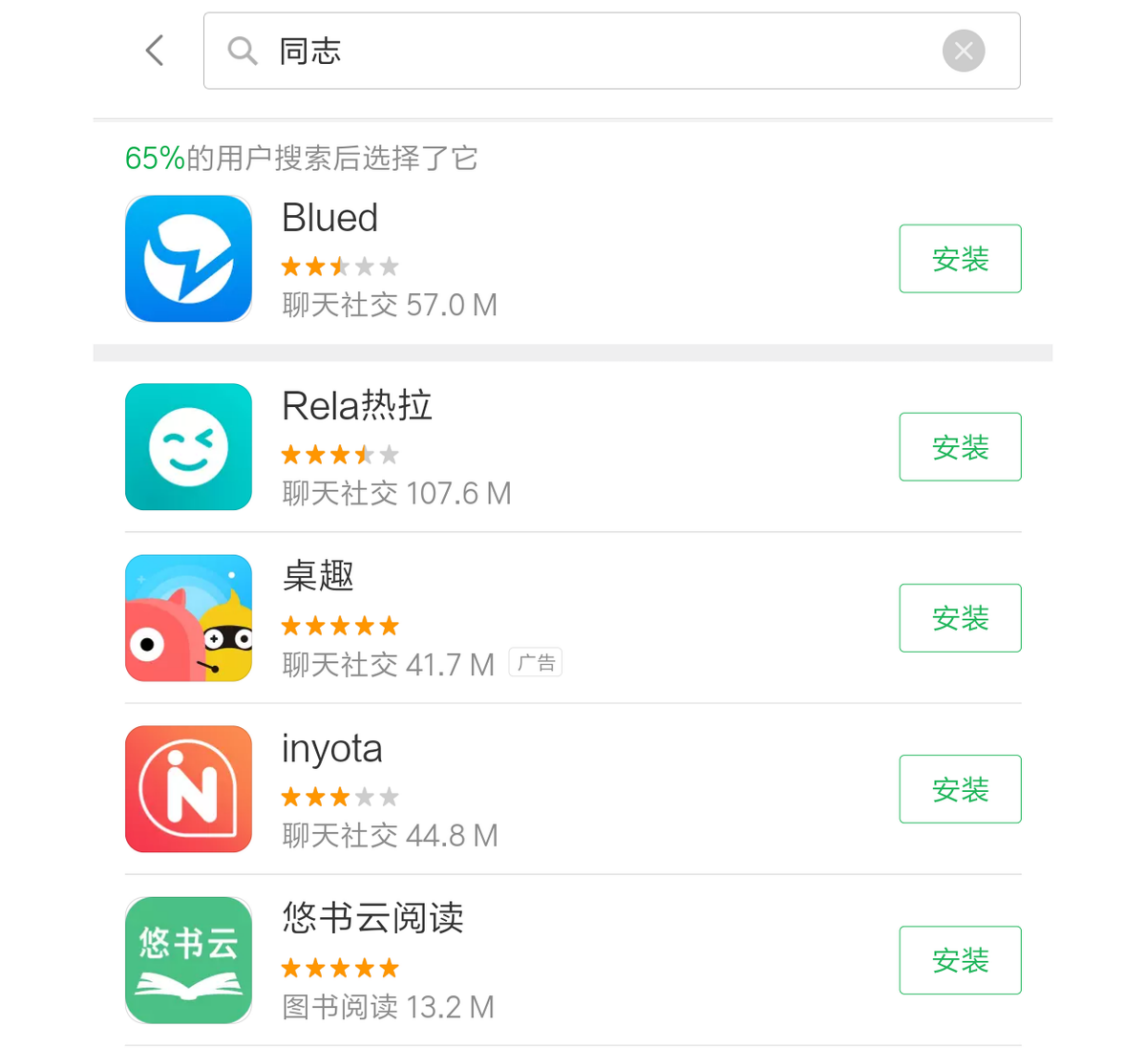
Some examples of MSM Apps for Android.

During the period between September 15, 2018 and September 30, 2018, the search in the Apple store and Android Market identified 382 and 193 apps, respectively (Figure 1). After reviewing the app titles and the app descriptions, 48 met the inclusion criteria. These 48 apps were then downloaded. However, 5 of them could not be opened or logged in. Therefore, they were excluded. Finally, 43 apps were reviewed. Some examples of MSM apps for iPhone and Android have been provided in Figures 2 and 3.

### Review Process

The review process comprised 2 steps. First, basic information about the 43 eligible apps, such as app name, app store category, and date of last update was gathered from the 2 specified app stores. Apps were categorized into groups on the basis of their primary target groups. Popularity is a quantity related to the number of users who have installed and are currently using the software app [17]. There are many metrics indicating an app’s popularity. This study chose the most commonly cited metrics: user rating and number of downloads [18]. These were extracted directly from the app stores if available. Second, all the 43 eligible apps were installed in a phone (Android or Apple). The Mobile App Rating Scale (MARS) was used to assess the apps’ quality. There are 2 versions of MARS: 1 evaluates apps from the researchers’ perceptive [19], whereas the other from the app users’ perspective [20]. This study chose the MARS for researchers as it is suitable for checking whether there was health information contained in these apps that could compromise users’ health and safety. Besides, the MARS for researchers provides a deeper evaluation of the quality of the health-related apps by testing them thoroughly for 10 min. This scale has been used in a similar app review, and it yielded good reliability and validity [21-23]. It includes objective quality and subjective quality. Objective quality has 4 domains including engagement, functionality, esthetics, and information. Each item in this 23-item scale is scored using a 5-point scale (1=Inadequate, 2=Poor, 3=Acceptable, 4=Good, and 5=Excellent). A cut-off of 3.0 has been previously identified as a minimum acceptable score [23].

Furthermore, 2 researchers independently assessed the 43 eligible apps using the MARS for researchers. Moreover, 1 of them was a research officer with a Research Master’s degree in Psychology and 2 years of experience in mobile app development, and the other was a PhD candidate with a Master’s degree in Nursing and over 6 years of experience in HIV-related research. These 2 researchers were first trained using Web-based video training resources to ensure that they correctly used the MARS scale. The 3 apps were used for the training and piloting purposes. All the 43 eligible apps were used for a minimum of 10 min when rating them. If there was any disagreement, a third reviewer would reassess it to achieve consensus, and interrater reliability was calculated.

### Statistical Analysis

All analyses were conducted using SPSS version 20.0 (SPSS Inc). Descriptive scores were calculated from the MARS scale. Intraclass correlation coefficient (ICC) among the raters was computed as a measure of interrater reliability for the MARS scale. A 2-way mixed, absolute agreement, average measures model was used to estimate the reliability of the average measures among the 2 raters.

## Results

### Overview

Among the 43 eligible apps, it was observed that 16 came from the Apple Store, 10 from the Android Market, and 17 from both the app stores. In addition, 3 apps needed to pay money for HIV test report verification when registering, whereas 40 apps were free to use. According to the 2 app stores, apps were categorized as social (n=29), life (n=5), entertainment (n=4), health and fitness (n=3), tool (n=1), and medical care (n=1). The primary target populations of the apps were gay (n=32), LGBT (n=5), gay or bisexual (n=3), gay or lesbian (n=2), and HIV-positive (n=1). Moreover, of the 43 eligible apps, 31 had been updated over the past year at the time of this study. In general, the apps were frequently downloaded, with 21 apps exceeding 10,000 downloads. Furthermore, 37 of the 43 eligible apps were rated by customers, yielding an average rating of 4 out of 5 stars. Moreover, 70% (26/37) were rated over 4 stars. Table 1 shows the details.

### Characteristic of the Apps

Overall, 39 out of the 43 eligible apps were developed as social network tools for finding friends and sexual partners. Examples of these apps are Blued, Jack’d, Gaypark, and Grindr. These are particularly gay apps, which allow users to filter people they wish to see by age, height, weight, and location. In addition, they provide various services, such as Web-based talking, sharing personal training or experience, news delivery, and watching television shows. On the contrary, only 4 apps (A health, Healthscore, RrainbowLaw, and pop) did not have the function for seeking sexual partners. In particular, *A health* is an app that provides self-management information for HIV-positive people, and it can provide HIV counseling for MSM. In addition, *Healthscore* app was designed specifically for MSM to provide information about sexual health and drug use, HIV counseling, checking of HIV-test results, and behavior assessment. Besides, *RrainbowLaw* app was designed to offer legal knowledge for LGBT, whereas *pop* app was aimed at promoting sexual health by encouraging users to adhere to safe-sex practices. In total, 10 apps (Blued, Rainbow rabbit, Healthscore, inyota, Blueboy, blueMr, homo, Bluefly, pop, and SMSM) contained sexual health information related to HIV/STDs, HIV testing, condom use, and links to Center for Disease Control and Prevention (CDC) or nongovernmental Organizations (NGOs). The name, target population, category, rating, update, and primary content areas of each eligible app are presented in Multimedia Appendix 1.

**Table 1:** Summary characteristics of men who have sex with men apps (N=43).

Characteristics of apps		Statistics, n (%)
**Phone platform**		
	Apple Store	16 (37)
	Android Market	10 (23)
	Both	17 (40)
**App store category**		
	Social	29 (68)
	Life	5 (12)
	Entertainment	4 (9)
	Health and fitness	3 (7)
	Medical care	1 (2)
	Tool	1 (2)
**Primary target population**		
	Gay	32 (74)
	LGBT^a^	5 (12)
	Gay or Bisexual	3 (7)
	Gay or Lesbian	2 (5)
	HIV-positive	1 (2)
**Updated over the past year**		
	Yes	31 (72)
	No	12 (28)
**Number of app downloads^b^**		
	0-999	3 (11)
	1000-9999	3 (11)
	>10,000	21 (78)
**User star rating**		
	N/A^c^	6 (14)
	1-3.9	11 (26)
	4-5	26 (60)

^a^LGBT: lesbian, gay, bisexual, and transgender.

^b^Information on number of app downloads is only available for apps in the Android Market (n=27).

^c^N/A: not applicable.

**Table 2:** Descriptive results of Mobile App Rating Scale scores (N=43).

Quality	Rater 1, mean (SD)	Rater 2, mean (SD)	ICC^a^ (95% CI)
Engagement	3.21 (0.71)	3.17 (0.69)	.834 (0.715-0.906)
Functionality	3.78 (0.69)	3.64 (0.79)	.766 (0.609-0.865)
Esthetic	3.25 (0.95)	3.22 (0.96)	.841 (0.726-0.910)
Information	3.31 (0.66)	3.28 (0.77)	.792 (0.650-0.881)
App quality	3.41 (0.67)	3.23 (0.64)	.946 (0.904-0.970)
Subjective quality	2.95 (0.98)	2.98 (0.99)	.910 (0.841-0.950)

^a^ICC: Intraclass correlation coefficient.

### Quality of the Apps

The quality of most apps (72%, 31/43) was identified as acceptable (MARS score>3). Functionality was the highest scoring domain, followed by information quality, esthetics, and engagement. In all, 3 apps (Blued, Gomeet, and Gtalk) achieved a score of 4.0 or more on both the overall quality and subjective scales, indicating high or excellent quality [23]. Interrater reliability was excellent for the overall mean app quality scores (ICC=.946, 95% CI 0.904-0.970) and the subjective quality scores (ICC=.910, 95% CI 0.841-0.950). Table 2 shows the details. Raw data of MARS scores are available in Multimedia Appendix 2.

## Discussion

### Principal Findings

This review assessed the characteristics and quality of 43 MSM apps in China, and it assessed whether these apps disseminated health information. With regard to the characteristics, a majority of the MSM apps (39/43) are used for social and sexual networking. The mobile app, Grindr, was released in 2009 and was the first to allow gay men to find potential sex partners nearby by using Global Positioning System (GPS) functions [24]. More apps have since been launched. Mobile phone apps have become the most common way for gay men to meet male sex partners. In this study, acceptability and popularity of MSM apps were roughly measured by users’ ratings and number of downloads. Usually, apps that are downloaded far more frequently have high user rating. Thus, Blued, Aloha, and Gaypark were found to be downloaded more than 100,000 times from the Android Market, and their rating was over 4 stars. Although Jack’d is considered to be the world’s fastest growing gay mobile app, the Chinese version of Jack’d was rated 2.5 stars by Chinese users even though it had a high number of downloads. The reasons why some international gay apps are rated poorly in China could be that they are translated versions of the international gay apps, and this might not fully cater to Chinese users’ cultural characters; therefore, they may achieve lesser satisfaction among the MSM in China when compared with the MSM using original versions outside China. Nonetheless, measuring acceptability and popularity of apps using the number of downloads and users’ ratings could lead to bias when the number of users is small. Therefore, it is important to highlight that there are many ways to measure and quantify the popularity using different metrics, namely chart rankings, user ratings and user reviews, downloads, the time spent by users on the mobile app, and the period between the installation of a mobile app and its removal from the user’s phone [18]. The popularity information of mobile apps often varies frequently and has the instinct of sequence dependence [18]. It is not possible to analyze whether the app is really popular or not. Yet, user rating and number of downloads are useful to guide mobile app selection for users.

In general, the majority of the MSM apps (31/43) have acceptable quality in relation to the apps’ characteristics, such as functionality, esthetics, usability, ability to engage users, and quality of information provided. Only 3 of 43 apps were identified as high quality. Features of high-quality apps include more engagement, multifunction, clear navigation, and layouts. As expected, high-quality apps had a higher average number of users and higher rating scores. As the main purpose of app developers is to make money, this suggests that developers have responded to users’ preference for functionality and tried to attract and retain more users by exquisite design and function expansion [25,26]. In terms of MARS subscales, engagement scored the worst, especially the item about interactive function. Interactivity is defined within the parameters of user input, feedback, prompts, virtual rewards, Web-based-offline integration, and family involvement [27]. Well-designed apps allow the users to choose which app features they wish to use and select messages and notifications they wish to receive through the app. The interactive functions of reviewed apps in this study are available but limited. This leads to many of the functional features, like providing intervention and care services that cannot be covered in apps.

Apps offer convenience to both the users and the developers as they provide a flexible way to reach a large audience at an affordable cost [28]. Several studies have demonstrated that young adults consider sexual health promotion via apps acceptable [25,28]. Sun et al reported that approximately two-third of MSM were willing to receive sexual health–related information through apps, and 26% of them requested referrals for HIV and STD testing [29]. In this study, 10 apps contain information about HIV testing and sexual health promotion. There are 4 modes to display sexual health content: forum, blog posts, infection status in users’ profiles, and links to sexual health information in the apps or linked websites. Each of these modes has its own limitations in reminding users of their sexual health. First, in-app blog posts or forum can be good places to display information regarding HIV/STDs if this information is correct and updated frequently. Second, having HIV status on a profile can be a good way to assist users filter partners by HIV status. Apps (Bluefly, BlueMr, and Homo) require users to upload their HIV test reports when registering in these apps and pay some money (about 20 RMB) for verifying their reports. The advantage of integrating HIV testing report into apps is to promote HIV testing if MSM want to use these apps. MSM may feel safe to make friends through these apps. However, there are still some concerns. For example, potential stigma will be produced if MSM are HIV-positive, and information safety may be questioned by users. Third, some apps offer links to CDC or NGOs such that only the address and telephone number of these institutions can be found in the apps. Therefore, this might be less effective as users have to be actively looking for sexual health information, which is not provided on the contact information of these institutions in the apps.

Unfortunately, this study found that only 1 out of the 43 MSM apps had information about drug use despite the fact that drug use is common among MSM. Zhao et al found that 77.30% (1100/1424) of the MSM subjects reported ever using recreational drugs in their lifetime, and poppers are the most popularly used among MSM [30]. As popper users tend to have more sexual partners [31], gay apps could facilitate the organization of private sex parties involving recreational drug use [32]. The combined recreational drug use and gay apps may create a virtual risk environment for HIV transmission among MSM in China [30]. More work needs to be done to increase awareness of the risk of drug use via MSM apps.

### Implication

MSM are disproportionately affected by HIV domestically and globally, and they are a key population for HIV infection and STDs prevention [33,34]. On the basis of existing evidence, the utilization of interactive Web-based and smartphone technologies to deliver sexual health information to MSM has shown promise. We suggest that public health researchers and app developers work together to promote sexual health through the existing popular MSM apps. For example, this can be realized by targeting and collaborating with some most frequently used MSM apps when advertising the beneﬁts of prevention and intervention programs for MSM populations. Health information should be displayed in various ways to increase attraction, such as pop-up messages, blog post, webcast, forum, and links to sexual health information. In addition, it is possible for health providers to use GPS data from the apps and provide services according to users’ physical locations, such as referral to HIV-testing centers. Exposing users to sexual health content when they are using these apps might be a good way to remind them of safe-sex practices. The impact of these messages on users’ behavior and health outcomes needs to be explored in further studies. Nonetheless, it should be highlighted that certain subgroups of MSM in China still preferentially meet at these venues (eg, bathhouse, pub, or club). Venue-based interventions such as on-site provision of volunteer testing and counseling and HIV awareness and knowledge promotion should also be emphasized.

In China, there is a lack of standards for app auditing. The less rigorous audit system leads to uneven quality of app products. The policy makers should establish the platform for supervision and audit and improve the legal system to regulate smartphone apps. For instance, when developing new apps, the developers should integrate health information into apps, especially for HIV high-risk populations. Establishing relevant regulations could guard the sexual health of MSM app users.

### Limitations

There are some limitations in this study. First, data collection only took place between September 15, 2018 and September 30, 2018. This means that the findings of this study could only provide a snapshot of the rapidly developing app market. MSM apps are changing rapidly; therefore, our search results might be different if repeated. Second, the quality of apps was assessed by MARS for researchers. The result might be different if assessed by the apps’ users. Third, there are many Chinese words that can be translated into the term *gay* but not all of them were used in our search for the MSM apps. Therefore, the search terms used were not exhaustive. Finally, our search was limited to the Apple Store and the Android Market; therefore, our search neglected apps from other smartphone operating systems (eg, Microsoft, Symbian, and BlackBerry).

### Conclusions

By reviewing the available apps, we found that MSM apps are popular. A majority of them are used for dating. Limited apps contain HIV prevention- and health-related information. The overall quality of the MSM apps is acceptable. MSM is a hidden population and a key population for HIV/STDs infection and prevention in China. Due to low rate of integration of sexual health information in gay apps, we suggest that public health researchers and app developers work together to promote sexual health through the existing popular MSM apps, which have the potential to increase HIV testing and linkages to appropriate care. The policy makers should establish the platform for supervision and audit to promote the health of the Chinese MSM app market.

## Appendix

Multimedia Appendix 1 List of men who have sex with men apps.

Multimedia Appendix 2 Raw data of Mobile App Rating Scale.

## Abbreviations

CDC: Center for Disease Control and Prevention
GPS: Global Positioning System
ICC: intraclass correlation coefficient
LGBT: lesbian, gay, bisexual, and transgender
MARS: Mobile App Rating Scale
MSM: men who have sex with men
NGOs: nongovernmental organizations
STDs: sexually transmitted diseases
