# Supplementary material for: The Characteristics and Quality of Mobile Phone Apps Targeted at Men Who Have Sex With Men in China: A Window of Opportunity for Health Information Dissemination?
Source: JMIR Mhealth Uhealth. 2019 Mar 27;7(3):e12573. doi: 10.2196/12573 (PMC6456822; doi:10.2196/12573)
Supplement: Multimedia Appendix 1 [file mhealth_v7i3e12573_app1.docx]

Detail information of reviewed MSM apps (N=43)

| Apps name | Target population | Rating | APP  focus on | Other information | | Update^#^ | Category |
| --- | --- | --- | --- | --- | --- | --- | --- |
|  |  |  | Dating | HIV testing | Sexual health |  |  |
| Platform  Both |  |  |  |  |  |  |  |
| Blued ^c^ | gay | 5 | Y | Y | N | Y | social |
| Jack’d ^c^ | gay | 2.5 | Y | N | N | Y | social |
| Aloha ^c^ | gay | 4.5 | Y | N | N | Y | social |
| Turn over the brand ^c^ | gay/lesbian | 4.5 | Y | N | N | Y | social |
| hornet ^c^ | gay | 4 | Y | N | N | Y | social |
| Rainbow rabbit ^c^ | gay | 4 | Y | Y | Y | Y | tool |
| Grindr ^c^ | gay/bisexual | 2.5 | Y | N | N | Y | social |
| boyAhoy ^c^ | gay | 3.5 | Y | N | N | Y | social |
| Lump sugar entertainment^c^ | gay | 4.5 | Y | N | N | Y | entertainment |
| Surge ^b^ | gay | 4.5 | Y | N | N | Y | life |
| A health ^b^ | HIV-positive | 4 | N | Y | Y | N | Medical |
| Gomeet^a^ | LGBT | 3.5 | Y | N | N | N | social |
| Healthscore ^a^ | gay | - | N | Y | Y | N | health & fitness |
| Cherry gay ^c^ | LGBT | 2 | Y | N | N | N | social |
| BlueG ^c^ | gay | 5 | Y | N | N | Y | life |
| Pepper gay ^c^ | gay | 5 | Y | N | N | Y | health & fitness |
| inyota c | gay | 4.5 | Y | Y | Y | Y | social |
| Android market |  |  |  |  |  |  |  |
| Wanwan ^a^ | gay | - | Y | N | N | N | social |
| Gaypark ^c^ | gay | 4 | Y | N | N | N | social |
| Friend G ^c^ | gay | 3 | Y | N | N | N | social |
| Blueboy ^c^ | gay | 4.5 | Y | Y | N | Y | life |
| blueMr ^c^ | gay | 5 | Y | Y | N | Y | life |
| Soguy ^c^ | gay | 2 | Y | N | N | N | social |
| rainbow fate ^c^ | gay/lesbian | 3.5 | Y | N | N | N | social |
| Skyboy ^b^ | gay | 3.5 | Y | N | N | Y | social |
| Homo ^c^ | gay | 4.5 | Y | Y | Y | Y | social |
| Bluefly ^c^ | gay | 4.5 | Y | Y | Y | Y | social |
| Apple store |  |  |  |  |  |  |  |
| Pull bear | gay | 2.5 | Y | N | N | N | social |
| fridae | LGBT | 4.5 | Y | N | N | Y | social |
| Buddy | gay | - | Y | N | N | Y | social |
| wapo | gay/bisexual | 5 | Y | N | N | Y | social |
| He | gay | 5 | Y | N | N | Y | social |
| blueman | gay | 2 | Y | N | N | Y | social |
| guyspy | gay/bisexual | 4.5 | Y | N | N | Y | social |
| shuggr | gay | 4.5 | Y | N | N | Y | social |
| hotmale | gay | - | Y | N | N | Y | social |
| pop | gay | - | N | Y | Y | N | health & fitness |
| RrainbowLaw | LGBT | - | N | N | N | N | life |
| Chance | gay | 4.5 | Y | N | N | Y | entertainment |
| Gay talk | gay | 5 | Y | N | N | Y | social |
| Fly talk | gay | 5 | Y | N | N | Y | entertainment |
| Gtalk | gay | 5 | Y | N | N | Y | entertainment |
| SMSM | LGBT | 5 | Y | Y | Y | Y | social |

^a^ number of app downloads ranges from 0 to 999; ^b^ number of app downloads ranges from 1000 to 9999; ^c^ number of app downloads is over 10,000.

-: not available

Update^#^: update during the last year

Y: yes; N: no

LGBT: Lesbian, gay, bisexual, transgender
