# Supplementary material for: The Characteristics and Quality of Mobile Phone Apps Targeted at Men Who Have Sex With Men in China: A Window of Opportunity for Health Information Dissemination?
Source: JMIR Mhealth Uhealth. 2019 Mar 27;7(3):e12573. doi: 10.2196/12573 (PMC6456822; doi:10.2196/12573)
Supplement: Multimedia Appendix 2 [file mhealth_v7i3e12573_app2.docx]

MARS quality scores for MSM apps (N=43)

| App name | Engagement | | Functionality | | Aesthetic | | Information | | Subjective score | |
| --- | --- | --- | --- | --- | --- | --- | --- | --- | --- | --- |
|  | Rater  1 | Rater 2 | Rater  1 | Rater 2 | Rater 1 | Rater 2 | Rater 1 | Rater 2 | Rater  1 | Rater  2 |
| Blued | 4.20 | 4.00 | 4.50 | 4.00 | 4.33 | 4.30 | 3.71 | 4.00 | 4.50 | 4.50 |
| Jack’d | 2.20 | 3.20 | 3.00 | 3.60 | 2.33 | 3.00 | 2.40 | 2.00 | 1.50 | 2.00 |
| Aloha | 3.60 | 3.00 | 4.00 | 4.00 | 4.00 | 4.00 | 3.75 | 3.71 | 3.75 | 4.00 |
| Turn over the brand | 3.00 | 3.00 | 3.75 | 3.00 | 3.33 | 3.33 | 2.66 | 3.20 | 2.00 | 2.50 |
| hornet | 2.20 | 2.00 | 3.00 | 2.60 | 2.33 | 2.00 | 2.40 | 3.00 | 1.50 | 2.00 |
| Rainbow rabbit | 3.20 | 3.00 | 4.00 | 3.80 | 3.00 | 3.66 | 3.75 | 4.00 | 3.48 | 3.75 |
| Grindr | 2.20 | 2.60 | 3.00 | 4.00 | 2.33 | 2.00 | 2.40 | 2.66 | 1.50 | 1.50 |
| boyAhoy | 3.60 | 3.40 | 4.75 | 4.00 | 4.30 | 4.00 | 3.50 | 3.75 | 3.50 | 3.00 |
| Lump sugar entertainment | 3.80 | 3.60 | 4.00 | 3.60 | 3.66 | 4.00 | 3.50 | 3.71 | 3.75 | 3.25 |
| Surge | 2.50 | 2.50 | 3.00 | 2.80 | 3.66 | 4.00 | 4.00 | 3.50 | 3.00 | 3.00 |
| A health | 3.40 | 3.60 | 4.00 | 3.40 | 3.33 | 3.00 | 4.00 | 4.00 | 3.75 | 3.25 |
| Gomeet | 4.00 | 3.80 | 4.50 | 4.00 | 4.33 | 4.00 | 3.50 | 2.67 | 4.00 | 4.00 |
| Healthscore | 3.80 | 3.60 | 4.00 | 4.50 | 3.33 | 2.33 | 4.00 | 4.60 | 4.00 | 4.00 |
| Cherry gay | 2.50 | 2.80 | 3.00 | 2.00 | 3.66 | 3.00 | 3.75 | 4.00 | 3.22 | 3.25 |
| BlueG | 3.80 | 3.60 | 4.50 | 4.75 | 4.66 | 4.33 | 4.00 | 4.00 | 3.75 | 4.00 |
| Pepper gay | 3.60 | 4.00 | 3.50 | 3.75 | 3.0 | 3.33 | 2.67 | 3.00 | 3.75 | 4.00 |
| inyota | 3.40 | 3.20 | 3.75 | 4.00 | 3.33 | 3.00 | 3.60 | 3.20 | 3.50 | 3.00 |
| Wanwan | 2.60 | 2.00 | 3.25 | 3.00 | 3.00 | 3.66 | 2.50 | 2.75 | 2.00 | 3.00 |
| Gaypark | 2.80 | 2.60 | 4.00 | 4.20 | 3.66 | 3.33 | 4.60 | 4.00 | 2.50 | 1.50 |
| Friend G | 3.60 | 4.00 | 4.00 | 3.00 | 3.33 | 3.00 | 3.20 | 2.40 | 3.00 | 3.00 |
| Blueboy | 3.80 | 3.60 | 4.50 | 4.75 | 4.66 | 4.33 | 4.00 | 4.00 | 3.75 | 4.00 |
| blueMr | 3.80 | 3.00 | 4.50 | 4.50 | 4.66 | 4.00 | 4.00 | 4.60 | 3.75 | 3.75 |
| Soguy | 3.80 | 3.40 | 3.00 | 3.50 | 3.33 | 3.66 | 3.25 | 2.50 | 3.00 | 3.00 |
| Rainbow fate | 3.60 | 3.00 | 4.75 | 4.00 | 4.30 | 4.66 | 3.50 | 3.75 | 3.25 | 3.25 |
| Skyboy | 3.60 | 4.00 | 4.75 | 4.00 | 4.30 | 3.33 | 3.50 | 3.50 | 3.25 | 3.00 |
| Homo | 3.80 | 4.00 | 4.50 | 4.20 | 4.66 | 4.66 | 4.00 | 4.60 | 3.75 | 3.50 |
| Bluefly | 4.00 | 4.00 | 4.50 | 4.50 | 4.33 | 4.00 | 3.50 | 3.00 | 4.00 | 4.00 |
| Pull bear | 2.20 | 2.00 | 3.75 | 4.00 | 2.33 | 2.00 | 2.40 | 2.50 | 1.50 | 2.00 |
| fridae | 2.20 | 2.00 | 3.00 | 2.80 | 2.00 | 1.00 | 3.00 | 3.500 | 1.20 | 2.00 |
| Buddy | 2.00 | 2.40 | 2.00 | 2.00 | 1.00 | 1.00 | 1.75 | 2.50 | 1.00 | 1.50 |
| wapo | 2.80 | 2.40 | 3.00 | 3.60 | 2.33 | 1.60 | 2.50 | 2.50 | 1.50 | 1.00 |
| He | 3.40 | 2.60 | 2.75 | 3.00 | 3.33 | 4.00 | 2.75 | 2.50 | 3.05 | 3.50 |
| blueman | 4.00 | 4.00 | 4.50 | 4.50 | 4.33 | 4.00 | 3.50 | 3.00 | 4.00 | 4.00 |
| guyspy | 2.50 | 2.00 | 3.00 | 2.50 | 3.66 | 2.66 | 3.75 | 3.71 | 3.00 | 3.00 |
| shuggr | 2.60 | 3.00 | 2.75 | 2.00 | 2.00 | 2.00 | 2.67 | 1.75 | 2.00 | 1.00 |
| hotmale | 3.40 | 3.40 | 4.00 | 4.00 | 3.66 | 2.33 | 4.00 | 3.25 | 3.00 | 3.00 |
| pop | 2.80 | 2.80 | 3.00 | 3.00 | 2.33 | 2.00 | 2.50 | 2.50 | 1.50 | 1.00 |
| RainbowLaw | 1.20 | 2.00 | 3.50 | 2.00 | 1.00 | 2.00 | 2.30 | 2.00 | 1.00 | 1.00 |
| chance | 3.00 | 3.40 | 3.75 | 4.00 | 2.67 | 3.00 | 3.00 | 2.50 | 3.00 | 3.22 |
| Gay talk | 3.40 | 3.00 | 3.60 | 4.00 | 2.67 | 3.66 | 3.00 | 2.50 | 3.00 | 3.00 |
| Fly talk | 3.40 | 4.00 | 4.00 | 3.50 | 3.33 | 3.00 | 3.50 | 3.00 | 3.50 | 4.00 |
| Gtalk | 4.00 | 4.40 | 4.75 | 4.50 | 4.00 | 4.00 | 4.20 | 4.40 | 4.25 | 4.00 |
| SMSM | 4.20 | 4.00 | 4.25 | 4.50 | 3.33 | 3.00 | 3.60 | 4.20 | 3.00 | 3.50 |
